# Co-Exposure with Fullerene May Strengthen Health Effects of Organic Industrial Chemicals

**DOI:** 10.1371/journal.pone.0114490

**Published:** 2014-12-04

**Authors:** Maili Lehto, Topi Karilainen, Tomasz Róg, Oana Cramariuc, Esa Vanhala, Jarkko Tornaeus, Helena Taberman, Janne Jänis, Harri Alenius, Ilpo Vattulainen, Olli Laine

**Affiliations:** 1 Finnish Institute of Occupational Health, Helsinki, Finland; 2 Tampere University of Technology, Department of Physics, Tampere, Finland; 3 University of Eastern Finland, Department of Chemistry, Joensuu, Finland; 4 University of Southern Denmark, MEMPHYS – Center for Biomembrane Physics, Odense, Denmark; Jacobs University Bremen, Germany

## Abstract

*In vitro* toxicological studies together with atomistic molecular dynamics simulations show that occupational co-exposure with C_60_ fullerene may strengthen the health effects of organic industrial chemicals. The chemicals studied are acetophenone, benzaldehyde, benzyl alcohol, *m*-cresol, and toluene which can be used with fullerene as reagents or solvents in industrial processes. Potential co-exposure scenarios include a fullerene dust and organic chemical vapor, or a fullerene solution aerosolized in workplace air. Unfiltered and filtered mixtures of C_60_ and organic chemicals represent different co-exposure scenarios in *in vitro* studies where acute cytotoxicity and immunotoxicity of C_60_ and organic chemicals are tested together and alone by using human THP-1-derived macrophages. Statistically significant co-effects are observed for an unfiltered mixture of benzaldehyde and C_60_ that is more cytotoxic than benzaldehyde alone, and for a filtered mixture of *m*-cresol and C_60_ that is slightly less cytotoxic than *m*-cresol. Hydrophobicity of chemicals correlates with co-effects when secretion of pro-inflammatory cytokines IL-1β and TNF-α is considered. Complementary atomistic molecular dynamics simulations reveal that C_60_ co-aggregates with all chemicals in aqueous environment. Stable aggregates have a fullerene-rich core and a chemical-rich surface layer, and while essentially all C_60_ molecules aggregate together, a portion of organic molecules remains in water.

## Introduction

Fullerenes belong to carbon nanomaterials which are carbon allotropes first discovered in 1985 [1]. They have been under extensive research ever since and fullerene-based materials have already been used in numerous commercial applications [2]. Recent application areas include, e.g., cosmetics [3], electronics [4], and nanomedicine [5], which exploit the unique properties of fullerene such as high electron affinity and electron transfer capability as well as antioxidant and radical scavenging activities.

The wide exploitation of fullerene-based materials, especially in the foreseeable future, calls for attention to assess the occupational, environmental, and consumer safety of fullerene-based materials. In previous *in vivo* and *in vitro* studies [6]–[8], pure unmodified fullerenes have not shown any severe toxicological effects. The most severe findings have been explained by traces of tetrahydrofuran (THF) which is often used as solvent in preparation of stable fullerene suspensions for toxicological studies [9]–[11].

However, based on several examples, there are effects of fullerene exposure which are related to conditions where fullerene interacts with other compounds, either natural or synthetic, and the effects likely emerge from the properties of the self-assembled fullerene-based supramolecular complexes containing these compounds. The interactions between fullerene and other compounds may lead to changes in their bioavailability, bioaccumulation, transportation, and toxicity.

For instance, cells exposed to a mixture of C_70_ fullerene and gallic acid, a natural phenolic compound found in, e.g., tea and red wine, were observed to undergo cell death in about 15 minutes [12]. Another example includes an ecotoxicological study with algae and daphnids, where phenanthrene was more toxic with C_60_ fullerene than alone, whereas pentachlorophenol was less toxic to algae but more toxic to daphnids with C_60_ [13]. In the same study, bioavailability of all environmental pollutants decreased when they were associated with C_60_. In studies with 17α-ethinylestradiol and zebrafish, association with C_60_ also decreased the bioavailability of 17α-ethinylestradiol [14].

In addition, atrazine and polycyclic aromatic hydrocarbons (PAH) were adsorbed on C_60_ in aqueous environment [15], C_60_ increased the uptake of trichloroethylene by plants in a phytoremediation system [16], and C_60_ enhanced transportation of a polychlorinated biphenyl (PCB) compound and phenanthrene through sandy soil [17]. An *in vitro* study with zebrafish hepatocytes showed that C_60_ increased the uptake of arsenium but also reduced cellular injury caused by arsenium [18].

Our study is the first attempt to consider a potential hazard of occupational inhalation co-exposure to C_60_ and organic industrial chemicals. This kind of co-exposure might occur, for example, when fullerene dust and a vapor of organic chemicals are released simultaneously in workplace air, or when a fullerene solution is aerosolized therein. There is one earlier study with C_60_ and industrial chemicals where skin penetration of C_60_ in different industrial organic solvents was found more efficient with chloroform than with toluene or cyclohexane [19].

We studied acute cytotoxicity and immunotoxicity of C_60_ and five industrial chemicals, acetophenone, benzaldehyde, benzyl alcohol, *m*-cresol, and toluene, together and alone by using human THP-1-derived macrophages which are widely used models in *in vitro* nanotoxicology [20], [21]. Acute cytotoxicity was tested by measuring release of lactate dehydrogenase (LDH) and immunotoxicity was determined by measuring secretion of pro-inflammatory cytokines interleukin-1-beta (IL-1β) and tumor necrosis factor-alpha (TNF-α). *In vitro* studies were carried out with unfiltered and filtered aqueous mixtures of C_60_ and organic chemicals. The first ones represent co-exposure to fullerene dust and organic chemicals since they include also non-suspended fullerene particles and the latter ones represent exposure to aerosolized fullerene solution where fullerene is completely solubilized. Toxicological studies were complemented by atomistic molecular dynamics (MD) simulations on C_60_ and organic chemicals in aqueous environment.

## Materials and Methods

### Materials

Sublimed C_60_ fullerene (99.95%) was purchased from Materials Technologies Research (Cleveland, OH, USA) and organic chemicals including acetophenone (99%), benzaldehyde (>99%), benzyl alcohol (99%), *m*-cresol (99%), and toluene (99.9%) were obtained from Sigma-Aldrich (St. Louis, MO, USA).

### Preparation of Fullerene Suspensions

Suspension media (cRPMI) was made from RPMI 1640 culture medium (Gibco, Invitrogen, Paisley, UK) supplemented with 2 mM GlutaMAX 1 (BioWhittaker, Lonza, Verviers, Belgium), 10 mM HEPES (BioWhittaker), 50 µM β-mercaptoethanol (Sigma-Aldrich), 50 U mL^−1^ penicillin, and 50 µg mL^−1^ streptomycin. Fullerene stock suspension (1 mg mL^−1^) was prepared by dispersing fullerene in cRPMI containing 2% BSA (Sigma-Aldrich) and sonicating it in an Elma S15 H water-bath ultrasonicator (Elma & Co, Singen, Germany) for 20 minutes at 30°C.

The sonication step was repeated after further dilutions and suspensions were shaken overnight with or without organic chemicals prior to exposure to cells. Based on pretests, 200 µg mL^−1^ was the concentration of choice for fullerene, 10 mM for acetophenone, benzaldehyde, benzyl alcohol, and toluene, and 5 mM for *m*-cresol, in all toxicological studies. A part of the suspensions were filtered through a 0.45 µm filter (Millipore, Carrigtwohill, Ireland) prior to exposures.

### Fourier Transform Ion Cyclotron Resonance Mass Spectrometry

The purity and the exact mass of C_60_ fullerene were determined by using a Bruker Apex-Qe hybrid quadrupole-Fourier transform ion cyclotron resonance mass spectrometer (Bruker Daltonics, Billerica, MA, USA) with an Infinity ICR cell, a 12-T refrigerated superconducting magnet, and continuous flow electrospray ionization with an Apollo-II ion source. C_60_ fullerene was diluted to the concentration of 10 µM by using a mixture of toluene and acetonitrile (1∶1, v/v) as a solvent and the instrument was operated on negative mode.

### Dynamic Light Scattering and ζ-Potential Measurements

Intensity average diameters and ζ-potentials of C_60_ fullerene aggregates in suspensions used in toxicological studies were measured with a Zetasizer Nano ZS instrument (Malvern Instruments, Malvern, UK) which used DLS for size determinations, and laser Doppler velocimetry and phase analysis light scattering (PALS) for determination of ζ-potentials. The suspensions were filtered through a 0.45 µm filter before measurements and the analyses were accomplished at 37°C.

### Liquid Chromatography Mass Spectrometry

The concentration of C_60_ fullerene in the filtered suspension of pure fullerene was determined with a Micromass Quattro II triple quadrupole mass spectrometer (Manchester, UK) connected to an HP 1100 liquid chromatograph (Hewlett Packard, Waldbronn, Germany) with a Cosmosil 5PYE (4.6×150 mm, 5 µm) column and a Cosmosil 5PYE (4.6×10 mm, 5 µm) guard column (Nacalai Tesque, Kyoto, Japan). A mixture of toluene and methanol (6∶4, v/v) was used as an isocratic LC eluent at the flow rate of 700 µL min^−1^. Negative electrospray ionization was used as an ionization method and selected ion monitoring (SIM) for monitoring ions at *m/z* 720 and 734 for native and ^13^C-enriched C_60_ fullerene.

20–30% ^13^C-enriched C_60_ fullerene (500 ng, Materials and Electrochemical Research, Tucson, AZ, USA) was added to filtered samples as internal standard and fullerene was extracted from the samples with toluene. Toluene extracts were evaporated into dryness and reconstituted in the LC eluent. Quantification of C_60_ fullerene was based on the comparison of peak intensities of *m/z* 720 and 734 in SIM chromatograms. Calibration standards were prepared in the mixture of toluene and methanol (6∶4, v/v) at concentrations of 1–50 pg µL^−1^ which resulted in a linear calibration curve (*R* ^2^ = 0.999) presented in Fig. S1.

### Cell Cultures

A human monocytic leukemia cell line THP-1 was obtained from American Type Culture Collection (Manassas, VA, USA) and maintained in 10% FBS/cRPMI culture medium. THP-1 cells were differentiated into a macrophage-like phenotype by culturing on 6-well (1.5×106 cells/well) or 12-well (0.75×106 cells/well) plates (Corning, Lowell, MA, USA) for 48 hours in 10% FBS/cRPMI supplemented with 100 nM phorbol 12-myristate 13-acetate (PMA; Sigma-Aldrich) at 37°C in 5% CO_2_.

After PMA-differentiation, THP-1 cells were washed with Dulbecco’s phosphate-buffered saline without Ca^2+^ and Mg^2+^ (DPBS; Gibco) three times before stimulations with fullerene suspensions. Cell supernatants were collected, centrifuged (500 *g*, 5 min, 4°C), aliquoted, and stored at 7°C for subsequent cytotoxicity and at −70°C for cytokine assays. Cells were detached from the wells with Versene (Gibco) for transmission electron microscopy.

### Transmission Electron Microscopy

Cells were fixed with 2.5% glutaraldehyde in 0.1 M potassium phosphate buffer, pH 7.2 for 2 hours at room temperature, postfixed in 1% osmium tetroxide, dehydrated, and embedded in LX-112 resin (Ladd Research, Williston, VT, USA). Thin sections of cells were collected on uncoated copper grids, stained with uranyl acetate and lead citrate, examined with a JEM-1220 transmission electron microscope operating at an acceleration voltage of 100 kV (Jeol, Tokyo, Japan), and photographed with a Veleta TEM CCD camera (Olympus Soft Imaging Solutions, Münster, Germany).

### Cytotoxicity Assay

Acute cytotoxicity of different stimulants was measured from the cell culture supernatants by using an LDH assay according to instructions from the manufacturer (Roche Diagnostics, Mannheim, Germany). LDH release in the simulated samples was compared to that in the positive control and was presented as a percentage of LDH release of the positive control.

### Immunotoxicity Assay

Levels of pro-inflammatory cytokines IL-1β and TNF-α in the cell culture supernatants were determined after stimulations by a Bio-Plex 200 System (Bio-Rad Laboratories, Hercules, CA, USA) using Luminex xMAP technology and a Luminex xPONENT 3.1 software (Luminex, Austin, TX, USA) according to instructions from the manufacturer.

### Simulations and Model Systems

We considered nine model systems through 21 atomistic MD simulations which were complemented by free energy calculations for two systems (Table S1). All models contained 100 C_60_ and ∼85 000 water molecules placed in a cubic box with an edge length of 14 nm. Seven systems which were considered through atom-scale MD simulations contained 500 molecules of organic compounds, i.e. acetophenone, benzaldehyde, benzyl alcohol, *m*-cresol, or toluene. Three systems also contained NaCl at a physiological concentration of 120 mM.

**Figure 1:**
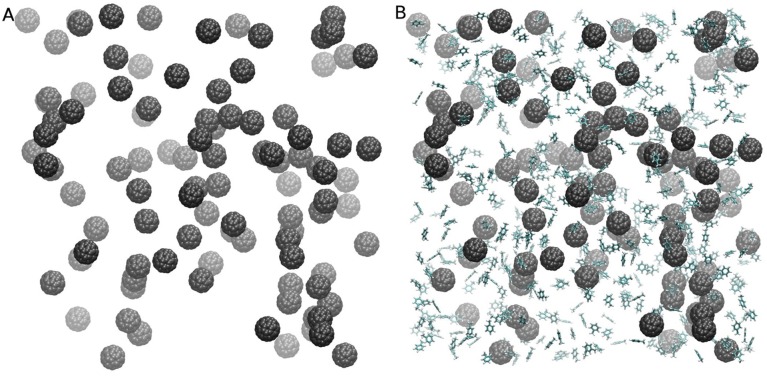
Initial configuration of the system composed of C_60_ (A) and C_60_ with toluene (B). Water molecules are not shown for clarity.

To improve sampling, three independent 100 ns simulations with randomly generated starting positions of both C_60_ fullerene and organic molecules were carried out for six systems (Table S1). Additionally, as discussed below, free energy calculations were carried out for two systems. The initial random structures of two systems after energy minimization with the steepest-descent algorithm are shown in Fig. 1.

We used the all-atom OPLS force field [22] for organic molecules and ions, and the TIP3P model compatible with OPLS parameterization [23] for water molecules. Bonded interactions determined from an x-ray study [24] and non-bonded interactions derived by Girifalco [25] were used for fullerene. This set of parameters was recently validated by Monticelli [26] for simulations of fullerene molecules in solution as well as in a complex with biological macromolecules [27].

Periodic boundary conditions were used in all three directions. The linear constraint solver (LINCS) algorithm [28] was used to preserve covalent bond lengths. The time step was set to 2 fs. The simulations were carried out at constant pressure (1 bar) and temperature (300 K). The temperature and pressure were controlled by using the Nose-Hoovér [29]–[30] thermostat and Parrinello-Rahman [31] barostat methods, respectively. The Lennard-Jones interactions were cut off at 1.0 nm and the particle mesh Ewald method [32] was employed for the electrostatic interactions. All MD simulations were performed with the GROMACS software package [33] over a time scale of 100 ns. This simulation protocol has successfully been used in our previous studies [34].

Umbrella sampling [35], [36] was used to estimate the free energy of translocation of a fullerene molecule from the cluster to the water phase. We first generated a radial pathway from the mass center of a fullerene cluster to the water phase (path length of 5 nm) by pulling out a central fullerene molecule. Next, the pathway was divided into windows separated by 0.1 nm in the cluster phase and 0.15 nm in the water phase. This resulted in a total of 53 windows for the C_60_/toluene mixture and 45 windows for pure C_60_. Force biased MD simulations were performed for 5.1 ns for each window (the cumulative simulation time was 315 ns) out of which 0.1 ns were considered as equilibration. The umbrella force constant was set to 1000 kJ mol^−1 ^nm^−2^.

The results obtained from all windows were combined using the weighted histogram analysis method (WHAM) [37] to provide the full thermodynamic evolution along the reaction coordinate, and the statistical error was estimated with a bootstrap analysis [38]. These calculations were performed for two systems, one composed of only C_60_ and another containing C_60_ and toluene (Table S1).

**Figure 2:**
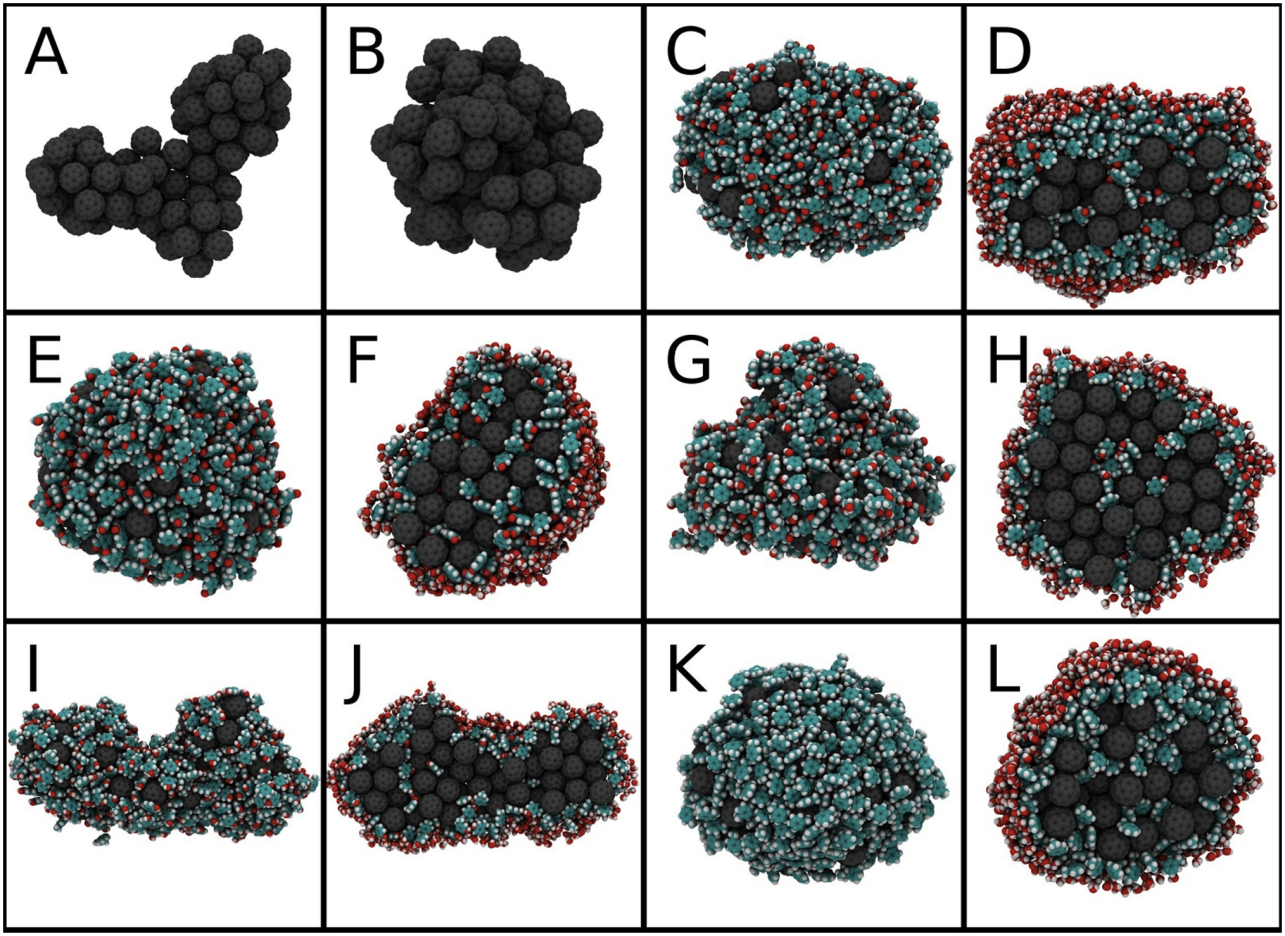
Final structures of C_60_ clusters with and without organic molecules. We use a color scheme for fullerene (gray), organic molecules (cyan/white/red for carbon/hydrogen/oxygen, respectively), and water (red/white). Pure C_60_ (A), spherical cluster of pure C_60_ for umbrella sampling (B), C_60_/acetophenone (C), C_60_/acetophenone cross-section (D), C_60_/benzaldehyde (E), C_60_/benzaldehyde cross-section (F), C_60_/benzyl alcohol (G), C_60_/benzyl alcohol cross-section (H), C_60_/*m*-cresol (I), C_60_/*m*-cresol cross-section (J), C_60_/toluene (K), and C_60_/toluene cross-section (L).

Because the cluster comprised of only C_60_ molecules was not spherical in the end of the simulation (Fig. 2) we constructed a spherical complex and equilibrated it for 0.5 ns prior to free energy calculations that were carried out with this system. For the mixture of C_60_ and toluene, we used the final structure obtained from the MD simulation. The center of mass position of the cluster was constrained over the entire length of the simulations. Figures were prepared with the VMD software [39].

### Analysis of Atomistic Simulations

To describe the aggregate in more quantitative terms we calculated RDFs of fullerene and organic molecules relative to the geometrical center of the cluster. RDF (*g_αβ_*) for a given distance *r* is defined in Equation 1:
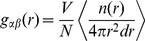
(1)where *n*(*r*) is the number of particles *β* in the spherical slice of radius *r* and width *dr* around the central particle *α* (geometrical center of the cluster in this case), 4π*r*2*dr* is the volume of the slice, and < > denotes an average over time and particles.

The percentages of organic molecules partitioning into the C_60_ cluster (Table 1) were calculated based on distance criteria. If an organic molecule was in contact with a molecule belonging to the cluster, i.e. the distance between any pair of atoms was smaller than 0.35 nm which is approximately the sum of van der Waals radii of two carbon atoms, this molecule was considered to be part of the cluster. Organic molecules in contact with water (a distance less than 0.35 nm) were recognized to be surface molecules. The remaining molecules were recognized as being located in the cluster core. This procedure was applied recursively.

**Table 1:** Partitioning of organic molecules in the simulated mixtures of C_60_ and organic chemicals.

Simulated mixture	Solvent	Organic molecules in solvent (*%*)	Organic molecules at the surface of the cluster (*%*)	Organic molecules in the core of the cluster (*%*)
C_60_+ acetophenone	Water	2.4±0.1	37.8±0.5	59.8±0.5
C_60_+ benzaldehyde	Water	5.3±0.6	40.5±0.9	54.1±1.1
C_60_+ benzyl alcohol	Water	24.4±0.4	44.6±0.2	31.0±0.5
C_60_+ benzyl alcohol	Saline	22.4	51.4	26.2
C_60_+ *m*-cresol	Water	13.6±1.0	51.8±2.4	34.7±1.6
C_60_+ toluene	Water	3.3±0.4	41.5±0.1	55.2±0.4
C_60_+ toluene	Saline	2.6	42.4	55.0

Each value is calculated as the mean from three independent simulations except for C_60_ and benzyl alcohol in water, for which there are two independent simulations, and for C_60_ with benzyl alcohol and toluene in saline, for which there is one simulation each. The error bars show the maximum deviation from the mean, calculated as the largest observed difference between an individual simulation and the mean.

### Statistics

The data were expressed as means ±SEM and were analyzed with GraphPad Prism 5 software (GraphPad Software, San Diego, CA, USA). Differences in toxicity of organic compounds with and without C_60_ were analyzed by using a Student’s *t*-test with a two-tailed test of significance and a Mann-Whitney *U*-test when variances were different between the groups for unpaired comparisons. Linear regression and Pearson correlation calculations were used to compare the results from the atomistic MD simulations and the toxicological studies. Differences at *p*<0.05 were considered to be statistically significant.

## Results

### Characterization of Fullerene and Fullerene Suspensions

Purity of C_60_ used in this study was confirmed by an ultra-high resolution mass spectrometric analysis with a Fourier transform ion cyclotron resonance mass spectrometer (FTICR-MS). The analysis did not show any signals of common impurities of C_60_ such as oxidized C_60_, C_70_, or other fullerene species. A mass spectrum from our C_60_ material is presented in Fig. S2.

Triplicate analysis results for intensity average diameters and ζ-potentials of C_60_ aggregates in filtered fullerene suspensions are presented in Table 2. Average diameters and ζ-potentials were not measured from unfiltered suspensions since the solid particles in those samples obstructed getting reliable results. Average diameters of aggregates measured by dynamic light scattering (DLS) did not differ significantly between the suspension of pure fullerene and those of fullerene with organic chemicals. The aggregate sizes in all suspensions ranged around 200 nm. ζ-potential of the pure C_60_ aggregate was a bit more positive than those of the aggregates with organic chemicals which did not differ from each other.

**Table 2:** Intensity average diameters and ζ-potentials of C_60_ aggregates in suspensions filtered through a 0.45 µm filter.

Suspension	Diameter (*nm*)	ζ-potential (*mV*)
C_60_	201±13	−8.9±0.3
C_60_+ acetophenone	227±15	−11.6±0.9
C_60_+ benzaldehyde	213±2	−13.1±0.9
C_60_+ benzyl alcohol	224±14	−12.6±1.4
C_60_+ *m*-cresol	196±23	−12.0±1.0
C_60_+ toluene	209±5	−12.5±1.2

Concentration of C_60_ in a filtered suspension of pure fullerene was determined by five parallel samples analyzed with liquid chromatography mass spectrometry (LC-MS). The concentration of C_60_ in the filtrates was 5.6±1.1 ng mL^−1^ which is only 0.028‰ of the C_60_ concentration in an unfiltered suspension.

### Cellular Uptake of Fullerene

**Figure 3:**
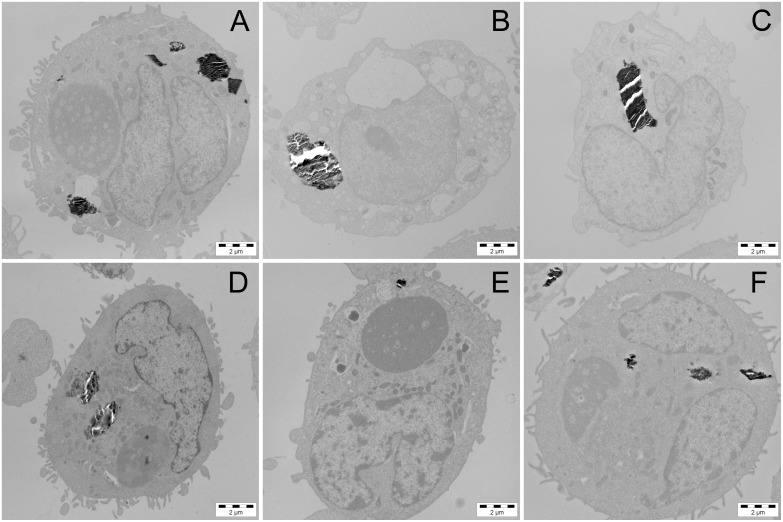
TEM images of human THP-1-derived macrophages. The cells were exposed to unfiltered suspensions of pure C_60_ (A) and C_60_ with acetophenone (B), benzaldehyde (C), benzyl alcohol (D), *m*-cresol (E), and toluene (F). Black deposits inside the cells represent aggregates of C_60_.

Transmission electron microscopy (TEM) imaging proved uptake of C_60_ by human THP-1-derived macrophages from C_60_ suspensions without and with organic chemicals. Fig. 3 represents TEM images of the cells exposed to unfiltered suspensions of pure C_60_ and C_60_ with organic chemicals. Based on the TEM images, C_60_ fullerene appeared inside the cells as large aggregates up to a diameter of 4 µm. Comparing this value to the aggregate size discussed in the previous section highlights that C_60_ aggregates with or without organic chemicals end up in cells, and the aggregate size increases by an order of magnitude.

### Acute Cytotoxicity of Fullerene Suspensions

Pretests with pure C_60_ and organic chemicals showed that all chemicals tested were acutely much more cytotoxic than C_60_ fullerene, encouraging us to study the influence of C_60_ on the toxicity of the chemicals. Based on pretests we selected the concentrations of C_60_ and organic chemicals used in cytotoxicity and immunotoxicity assays which resulted in 30–50% release of LDH compared to the positive control with organic chemicals, except for toluene, and 1–2% with C_60_ fullerene (data not shown).

LDH release with pure toluene was less than 1% which may be caused by evaporation of toluene during sample preparation and exposure to cells. Toluene is the most volatile chemical studied since its highest vapor pressure and concentration could not be increased because of its low water solubility. Physical constants of the chemicals studied are presented in Table 3. Our low-toxic concentration of C_60_ in *in vitro* studies corresponded to the high-level occupational inhalation exposure [7], [44].

**Table 3:** Dielectric constants, water solubilities, and vapor pressures of organic chemicals.

Organic molecule	Dielectric constant *ε*_r_ a)	Water solubility (*g L* ^−1^)^b, c)^	Vapor pressure (*Pa*)^d)^
Acetophenone	17.44	5.5^h)^	100^j)^
Benzaldehyde	17.85	3.0^f)^	100^e)^
Benzyl alcohol	11.92	35.0^f)^	10^i)^
*m*-cresol	12.44	23.5^f)^	1 ^g)^
Toluene	2.379	0.519^h)^	10 000^k)^

a) [40], ^b)^ [41], ^c)^ [42], ^d)^ [43], ^e)^19°C, ^f)^20°C, ^g)^21°C, ^h)^25°C, ^i)^28°C, ^j)^36°C, ^k)^45°C.

**Figure 4:**
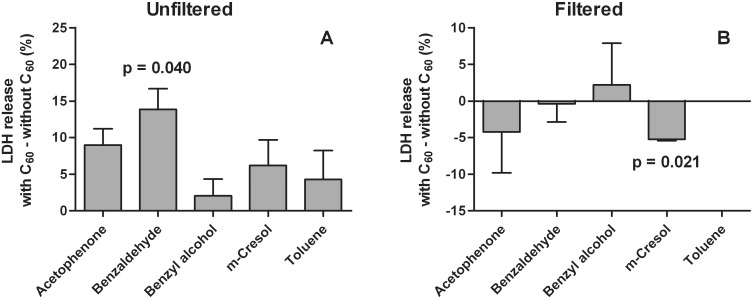
Differences in acute cytotoxicity between organic chemicals with and without C_60_. C_60_ was mixed overnight with and without organic chemicals in cRPMI medium containing 2% BSA before a 24 h exposure to the cells. Cytotoxicity was measured by LDH release compared to the positive control. Three unfiltered (A) and two filtered (B) samples were studied.

Fig. 4 depicts how C_60_ affects the cytotoxicity of organic chemicals. C_60_ slightly increased acute cytotoxicity of all chemicals in unfiltered suspensions but the co-effect was statistically significant only with benzaldehyde. An increase in cytotoxicity of benzaldehyde was 14% which was much more than the cytotoxicity of pure C_60_ fullerene. The positive co-effect in cytotoxicity of benzaldehyde disappeared with filtered suspensions. A statistically significant negative co-effect of 5% was observed with the filtered suspension with *m*-cresol which meant that it was less cytotoxic with C_60_ than alone.

### Immunotoxicity of Fullerene Suspensions

**Figure 5:**
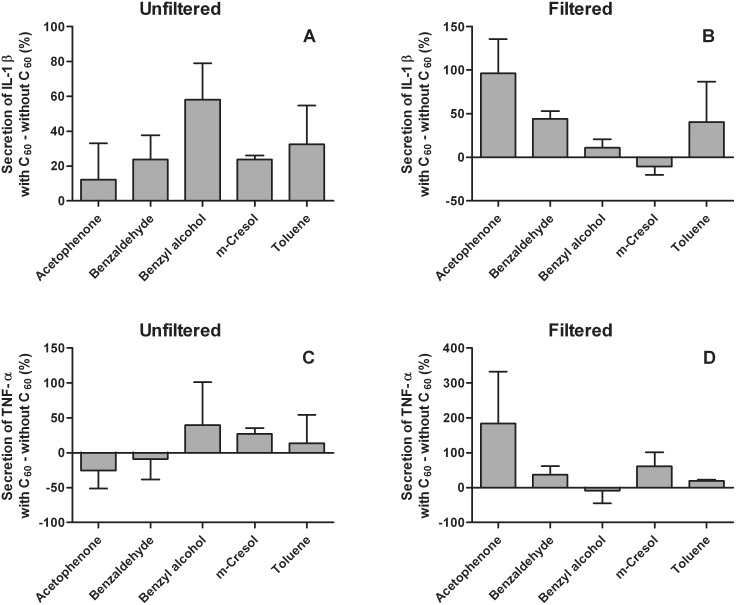
Relative differences in immunotoxicity between organic chemicals with and without C_60_. C_60_ was mixed overnight with and without organic chemicals in cRPMI medium containing 2% BSA before a 24 h exposure to the cells. Immunotoxicity was determined by measuring secretion of pro-inflammatory cytokines IL-1β (A, B) and TNF-α (C, D). Two unfiltered (A, C) and two filtered (B, D) samples were studied.

C_60_ fullerene did not cause any statistically significant co-effects in the immunotoxicity of organic chemicals. The co-effects in secretion of IL-1β and TNF-α are summarized in Fig. 5. The highest positive co-effects were seen with the same chemicals with both cytokines. The highest increase in secretion of IL-1β and TNF-α with C_60_ was observed with unfiltered suspensions with benzyl alcohol. The high positive co-effect with benzyl alcohol disappeared in filtered suspensions. The highest positive co-effect in the filtered suspensions was observed with acetophenone.

### Atomistic MD Simulations

Examples of initial configurations of simulated systems are shown in Fig. 1. Simulations indicated that fullerene and organic molecules aggregated rapidly, and in the end of the simulations the formed cluster included all fullerene molecules and a varying number of organic molecules. Only in the case of benzyl alcohol, more than one separate cluster remained after 100 ns in one out of three simulations. Two separate clusters of fullerene and benzyl alcohol remained stable for the whole duration of a 100 ns simulation. They did not merge after an additional 100 ns and were once able to approach each other without merging.

In the case of toluene, 98 mol-% of the molecules were involved in cluster formation, while with the most hydrophilic compounds up to 24 mol-% of the molecules remained in the water phase (Table 1). Complete aggregation of all fullerene molecules occurred in less than ∼66 ns in 80% of the systems comprised of organic molecules and C_60_ as well as those containing only C_60_. Sodium and chloride ions remained in the water phase and did not exert a noticeable influence on formation of the aggregate.

Snapshots of the clusters in the end of the simulations are shown in Fig. 2, highlighting that the clusters are composed of a central core region where fullerene dominates and the number of organic molecules is small, and of a shell region composed predominately of organic molecules. Except for the systems with benzyl alcohol or *m*-cresol, which contain individual water molecules in the shell region, no water is present in the clusters.

**Figure 6:**
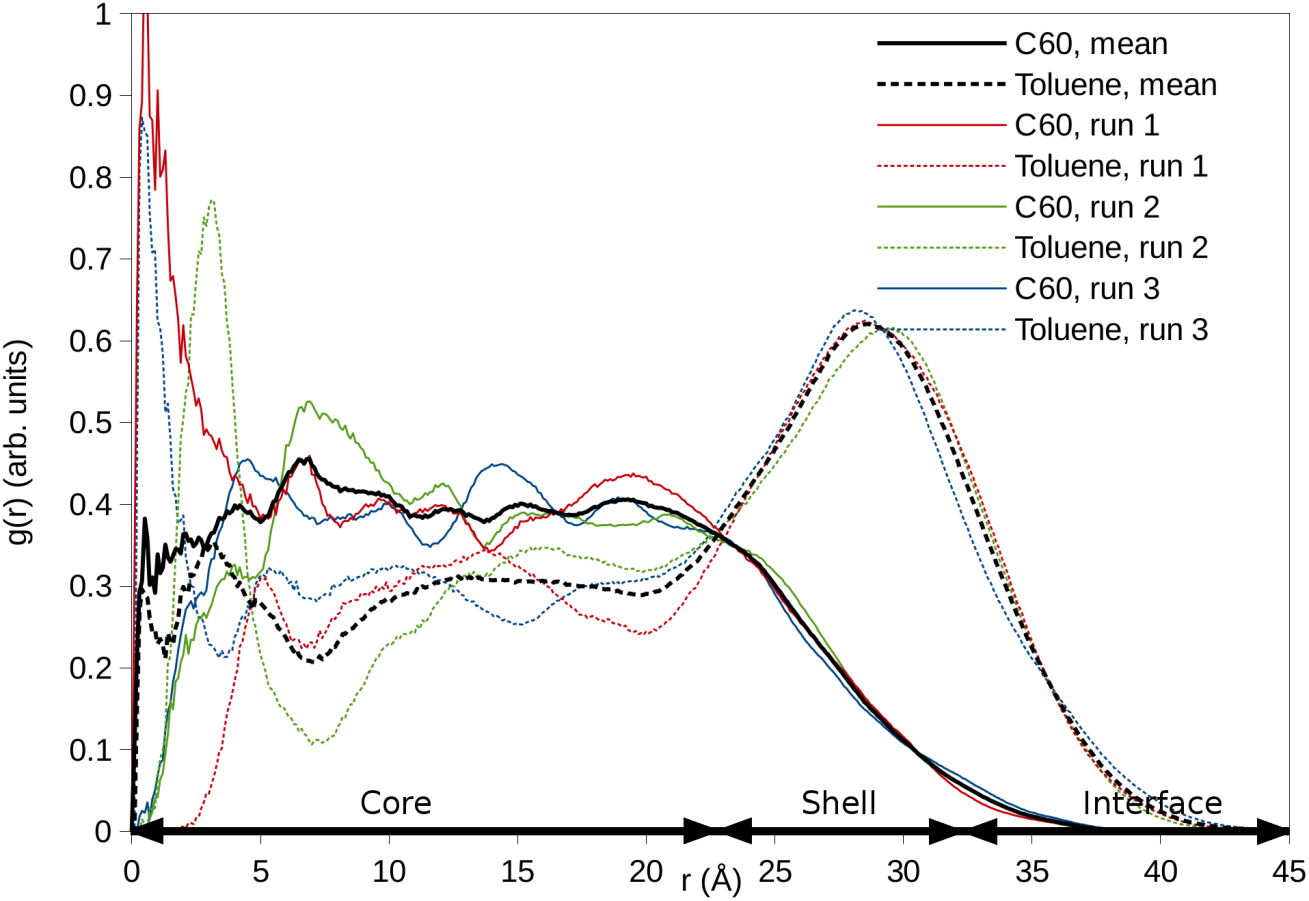
Radial distribution function (RDF) of C_60_ and toluene. RDFs are presented with respect to the geometrical center of the C_60_/toluene cluster. The core is defined as the region where C_60_ is the most abundant component, while the shell is the toluene-coating region around the core. The interface represents the contact region between the surface and water defined by the crossing point of the RDFs of water (not shown) and toluene.

The radial distribution function (RDF) of a C_60_/toluene cluster is shown as an example in Fig. 6. The other plots are given in Fig. S3. The RDFs demonstrate that in general the core region composed of fullerene and organic molecules has a radius of 1.5–2.5 nm. The shell region appears as a broad maximum in the RDF of organic molecules at a distance of ∼3 nm. It is evident that organic molecules not only reside inside the cluster but they also coat it, covering the surface of the aggregate. Toluene, acetophenone, and benzaldehyde form nearly spherical clusters with C_60_. The most hydrophilic compounds, benzyl alcohol and *m*-cresol, form more irregularly shaped clusters. The extent of the core and shell regions can be better defined for the spherically shaped clusters (Fig. 6 and S3), in which case also the interface region between the shell and water can be identified.

The shell composed of organic molecules around the C_60_ core is thin in systems with benzyl alcohol and *m*-cresol, thicker with acetophenone and benzaldehyde, and the thickest with toluene. Toluene is the least water-soluble compound of the simulated organic molecules, while benzyl alcohol and *m*-cresol are the most soluble ones. Additionally, there is a trace of sodium and chloride ions in the core of the cluster containing benzyl alcohol, and slightly more in the shell region (data not shown). However, most of the ions are distributed in the water phase. In the cluster with toluene, ions only appear at the outer edge of the toluene surface and none are found in the core. About 5–10% of the solvent-accessible surface (SAS) of co-aggregates consists of C_60_, while the remaining 90–95% are organic molecules. Table 1 further shows distribution of all organic molecules in simulated mixtures.

**Figure 7:**
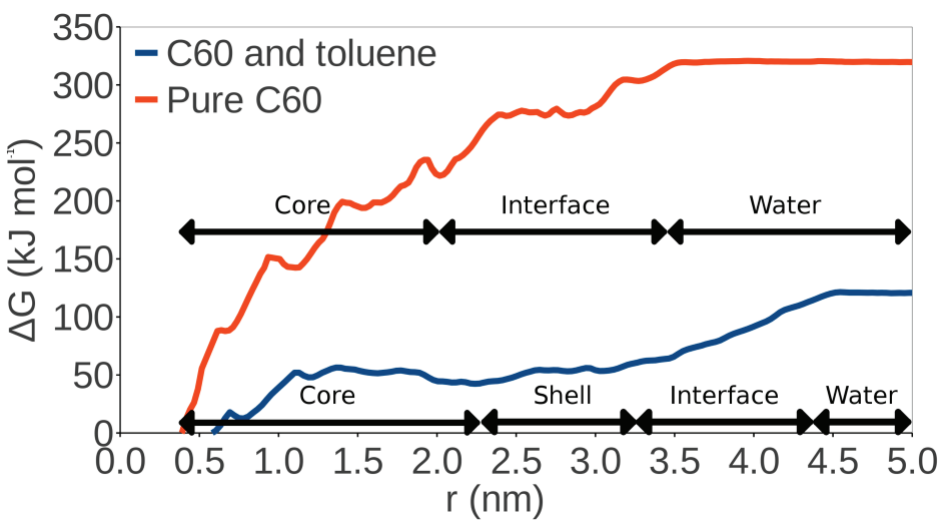
Change in free energy (Δ*G*) during the translocation of a single C_60_ molecule. Translocation is followed from the center of the cluster to the water phase along a radial distance *r*. The clusters are divided into various parts according to the RDF data. In the pure C_60_ cluster, the interface is where C_60_ and water are in contact.

Next, we used umbrella sampling simulations to determine the free energy change (Δ*G*) during the translocation of a single fullerene molecule from the center of the cluster to the water phase (Fig. 7). The free energy was set to zero in the cluster center. The obtained results indicate that the presence of toluene decreases the free energy of translocation by roughly a factor of three, from 320 to 120 kJ mol^−1^, indicating cluster stability weaken for increasing toluene concentration. Nonetheless, given these large numbers, it is clear that the considered clusters are very stable, being readily able to sustain thermal fluctuations. The bootstrap analysis gives error estimates of approximately 10 kJ mol^−1^ and 20 kJ mol^−1^ for the free energy calculations with and without toluene, respectively.

The rise of free energy in the beginning of the translocation process is very steep in the C_60_ system (Fig. 7) which correlates with the more compact aggregation of fullerene molecules in the core of the cluster as compared to the C_60_/toluene case. This is supported by the RDF data in Fig. S3.

Based on the free energy difference between C_60_ in an aggregate and C_60_ in water, and also on the dynamics in non-steered simulations, we conclude that the concentration of individual C_60_ molecules in solution is expected to be very small. Rather, C_60_ molecules are aggregated until they reach a radius of at least 4 nm (based on a simulation of pure C_60_ without organic molecules). Our simulations show that this is a lower bound for the size of C_60_ aggregates in solution. Thus, the observed biological effects cannot be explained by individual C_60_ molecules, but instead they arise from C_60_ aggregates, C_60_/organic molecule co-aggregates, or individual organic molecules.

### Correlation of the Results from Toxicological and Atomistic Simulation Studies

**Figure 8:**
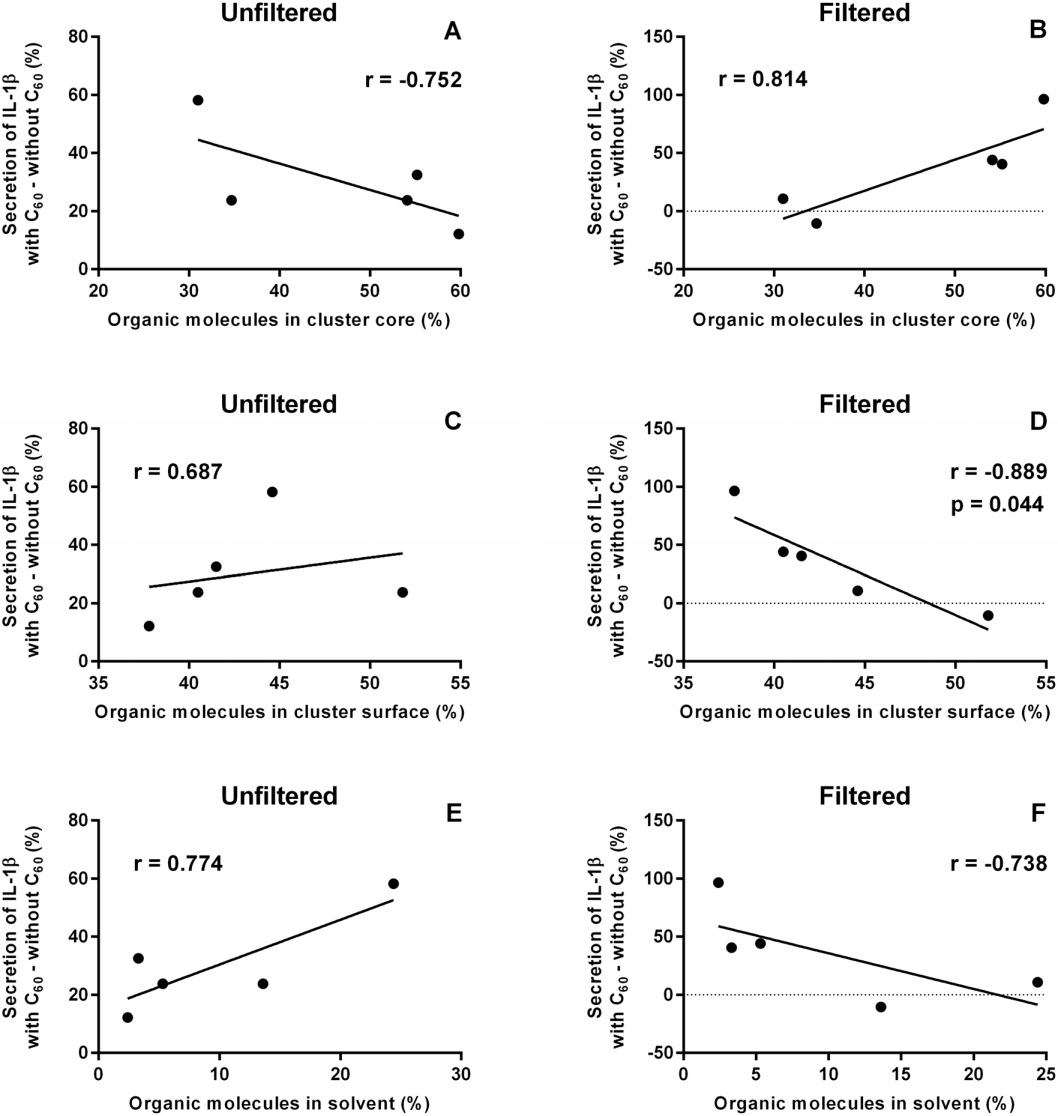
Correlation of C_60_-induced changes in secretion of pro-inflammatory cytokine IL-1β and partitioning of organic molecules. Correlations were determined for unfiltered (A, C, E) and filtered (B, D, F) samples, considering the partitioning of organic molecules in cluster core (A, B), cluster surface (C, D), and solvent (E, F) in the simulated mixtures.

**Figure 9:**
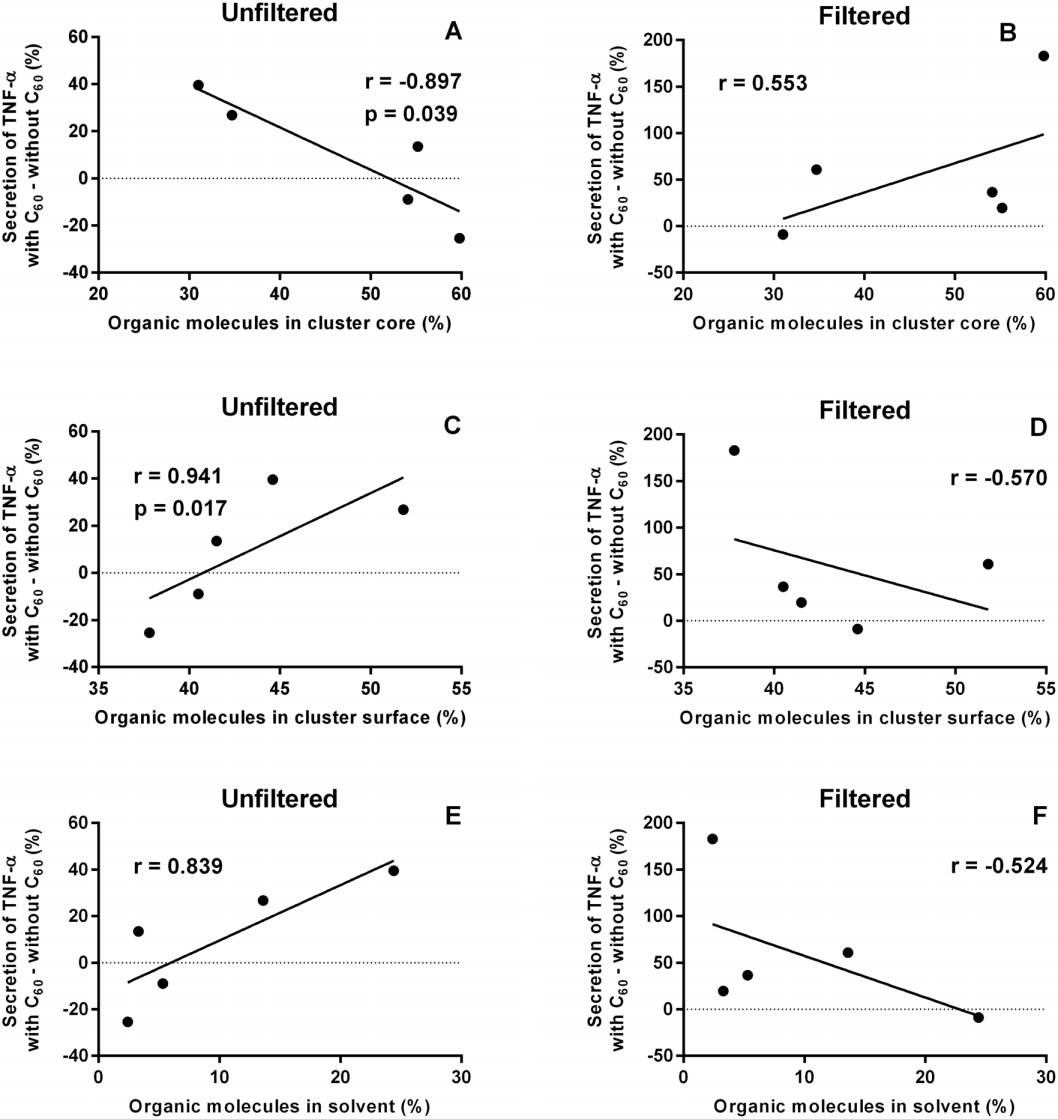
Correlation of C_60_-induced changes in secretion of pro-inflammatory cytokine TNF-α and partitioning of organic molecules. Correlations were determined for unfiltered (A, C, E) and filtered (B, D, F) samples, considering the partitioning of organic molecules in the cluster core (A, B), cluster surface (C, D), and solvent (E, F) in the simulated mixtures.

In order to evaluate correlations between the results from toxicological and atomistic simulation studies we viewed fullerene-induced changes in secretion of pro-inflammatory cytokines as a function of partitioning of organic molecules in the simulated mixtures. Fig. 8 and 9 show the correlations for IL-1β and TNF-α, respectively.

Fullerene-induced changes in secretion of IL-1β had a statistically significant negative correlation with the portion of organic molecules in cluster surface when the filtered suspensions were studied. In that case the highest increase in secretion of IL-1β was observed with acetophenone when there was the lowest portion of organic molecules at the cluster surface. In the same case, C_60_ hardly changed IL-1β secretion with benzyl alcohol and *m*-cresol when there were the highest portions of organic molecules at the surface of a cluster.

Other correlations with IL-1β were not statistically significant but there were some trends in IL-1β secretion and partitioning of organic molecules as follows: Trend lines of unfiltered and filtered samples were opposite to each other. The trend lines for organic molecules at the cluster surface and in solvent were similar but they were opposite to the trend lines for organic molecules in the cluster core.

Fullerene-induced changes in secretion of TNF-α significantly correlated with the portion of organic molecules in the cluster core and at the surface when the unfiltered suspensions were studied. In the cluster core the highest increase in secretion of TNF-α was observed with benzyl alcohol when there was the lowest portion of organic molecules in the cluster core. In the same case the greatest decrease in secretion of TNF-α was observed with acetophenone for the highest portion of organic molecules in the core.

At the cluster surface the highest increase in secretion of TNF-α was observed with benzyl alcohol and *m*-cresol for the highest portions of organic molecules at the cluster surface. Acetophenone resulted in the lowest share of molecules in the surface and the greatest decrease in secretion of TNF-α with C_60_, instead. All trend lines were similar to those for secretion of IL-1β but correlation coefficients of filtered samples were lower than in the case of IL-1β.

## Discussion

Results from our atomistic MD simulations agree with previous results from theoretical and experimental studies where highly hydrophobic C_60_ and C_70_ readily aggregate in water and also co-aggregate with organic molecules [12], [45]–[48]. Fullerene aggregates in aqueous fullerene suspensions prepared by a solvent-exchange method may contain traces of solvents used, even though solvents are evaporated from the suspensions which could have been proved by an FTIR spectroscopic analysis [46], [47], [49]. Based on current and earlier studies, C_60_ aggregates in the body very likely immediately after exposure when it comes into contact with physiological fluids.

Our simulations showed events where the organic molecule enters the fullerene aggregate, stays there, and is released back into the medium some tens of nanoseconds later. We also saw pockets of one or more organic molecules enclosed within C_60_ molecules which could have a long lifetime due to the strong reluctance of C_60_ to escape the clusters. On our timescale, C_60_ does not necessarily form spherical clusters but could form clusters with shaped surfaces.

In our simulations C_60_ also acted as a sponge which trapped, with our molar ratios, 75–98 mol-% of organic molecules from the solution in the aggregates. Once 90–95% of the C_60_ surface was covered by organic molecules, the remaining molecules could add another layer on top of the aggregate (hydrophobic toluene, acetophenone, and benzaldehyde), or remain in the solution (hydrophilic benzyl alcohol and *m*-cresol). A sufficiently high amount of C_60_ could remove all free organic molecules from the solution, whereas with a moderate amount of C_60_, a trace of free organic molecules was left in the medium.

Partitioning of different organic molecules in the simulated clusters can be explained mainly by water solubilities of the compounds studied. The most water-soluble compound was benzyl alcohol which was also the most abundant compound in solvent and the least abundant compound in the cluster core. Similarly the second most water-soluble compound *m*-cresol was the second most abundant compound in solvent, and the second least abundant compound in the core of a cluster. However, the least water-soluble compound toluene was not the most abundant compound in the cluster which was instead acetophenone. Abundances of acetophenone, benzaldehyde, and toluene in solvent were very close to each other. Polarity of the organic molecules, determined by dielectric constants, did not correlate with the partitioning behavior of the molecules in simulations since the most and the least polar molecules behaved similarly.

The aggregate sizes were similar to the aggregate sizes observed in earlier studies where C_60_ was dispersed in a solution containing fetal bovine serum (FBS) [50], [51]. ζ-potentials of the C_60_ aggregates with organic chemicals were also similar to those of the aggregates of FBS-dispersed C_60_ [51]. The ζ-potentials in all systems were quite small, suggesting that in suspensions the driving force for further aggregation is quite small beyond a size of ∼200 nm.

Water solubilities and dielectric constants of the organic molecules did not correlate with the measured average diameters and ζ-potentials of the C_60_ aggregates since these parameters remained practically unchanged when the organic molecules were changed. This may be explained by the protein corona which is very likely formed on the C_60_ aggregates in solutions containing bovine serum albumin (BSA) [51], [52]. Organic molecules may be partitioned differently in the pristine clusters, as the simulations suggest, but the protein corona covering the surface makes the clusters similar in terms of size and surface charge. This is also a likely situation in the body after an expected co-exposure on C_60_ and organic molecules. MD simulations on C_60_ aggregation with protein corona included are currently not feasible.

Our *in vitro* studies did not show as high co-effects in toxicity as ecotoxicological studies with C_60_ and environmental contaminants [13]. In an earlier ecotoxicological study, phenanthrene was 10 times more toxic to daphnids with C_60_ than without it, and pentachlorophenol was found to be almost two times less toxic to algae with C_60_ than without it. One reason for the magnitude of the co-effect might be the interaction time between C_60_ and organic molecules before exposure which was much shorter in our current work than in previous studies. In the study with algae and daphnids, C_60_ was first stirred alone in water for two months and then with organic pollutants for five days. In another study with zebrafish, C_60_ was first stirred alone in water for five months and then with 17α-ethinylestradiol up to 28 days [14].

Long stirring times of C_60_ in water simulate well situations when C_60_ fullerene is released in an ambient environment. Long mixing times of C_60_ with organic chemicals in aqueous solutions also enhance adsorption of organic molecules on fullerene particles and their co-aggregation. We wanted to keep the interaction time between C_60_ and organic chemicals short because we considered it to be the best way to simulate a situation in the body after occupational co-exposure. For the same reason we also wanted to study unfiltered suspensions of C_60_ fullerene instead of only filtered suspensions because they correspond to the situation when fullerene dust is inhaled.

Disappearance of slight positive co-effects in acute cytotoxicity after filtration of C_60_ fullerene suspensions can be explained by adsorption of organic molecules in fullerene clusters which are removed by filtration. The best example is benzaldehyde which is the second most hydrophobic and the second most abundant organic molecule in the simulated cluster core of the molecules studied. C_60_ may act as a carrier of organic molecules inside the cells as the novel fullerene-based drug delivery systems do which could cause an increased cytotoxicity of the organic compounds observed here [53].

However, the situation changes when the co-effects in immunotoxicity are considered. The highest positive co-effect in immunotoxicity with unfiltered suspensions is observed with benzyl alcohol which is the most hydrophilic compound studied. After filtration the highest co-effect is seen with poorly water-soluble acetophenone instead. It is possible that large non-suspended C_60_ particles together with the highly water-soluble compound are responsible for the highest positive immunological co-effects with unfiltered suspensions. Filtration removes large non-suspended particles, and the highest observed co-effect is then observed with the hydrophobic compound which is also highly abundant inside remaining small C_60_ aggregates.

Based on our current experiments, mechanisms behind differing immunological co-effects cannot be concluded but previous studies on diesel exhaust, carbon black, and ambient ultrafine particles also show that chemicals associated with particles may have various effects on the inflammatory response [54]–[56]. The study on human bronchial epithelial cells with exposure to diesel exhaust shows that removal of volatile and semi-volatile organic compounds from the exhaust which also consist of particles greatly reduce the release of interleukin 8 (IL-8) [54].

The study on human THP-1-derived macrophages with exposure to PAH compounds and carbon black particles together and alone shows opposite results since PAH does not change particle-derived secretion of IL-8 at all [55]. However, in the same study the secretion of IL-1β is much lower with PAH and ultrafine carbon black particles than with the same particles alone. In the study with mouse pulmonary endothelial cells co-exposure to aqueous cigarette smoke extract and ambient ultrafine particles gives rise to much higher expression of interleukin 6 (IL-6) than the both exposure agents alone [56].

Correlation analysis of C_60_-induced changes in secretion of pro-inflammatory cytokines and partitioning of organic molecules in simulated mixtures showed that abundance of molecules at the cluster surface and in solvent was linked to the co-effects in a similar way. The reason is probably that abundant molecules in solvent are also abundant at the cluster surface. The cluster surface is a dynamic environment where the molecules rapidly exchange with the molecules in solvent.

## Conclusions

Our study showed that co-exposure to organic industrial chemicals with C_60_ fullerene may strengthen the health effects of the chemicals. The co-effects observed here were quite minor, probably due to the short interaction time between the chemicals and C_60_ which simulates best the occupational co-exposure. Atomistic simulations were in agreement with experiments when comparison was appropriate. They showed that organic molecules readily co-aggregate with C_60_ fullerene in an aqueous environment. C_60_ clusters may contain more hydrophobic than hydrophilic organic molecules. Co-aggregation might explain the increased cytotoxicity of hydrophobic compounds in unfiltered fullerene suspensions but not the increased immunotoxicity of hydrophilic compounds in unfiltered suspensions. The results reported here stress the importance of being aware of different co-exposure scenarios at workplaces as co-exposure to fullerene together with other chemicals may pose effects on health. Our results can be applied also to environmental exposure to fullerene.

## Supporting Information

Figure S1 **A calibration curve for the LC-MS analysis of the C_60_ concentration.**
(TIF)

Figure S2 **An ultra-high resolution mass spectrum of the C_60_ material.**
(TIF)

Figure S3 **Radial distribution functions (RDFs) of C_60_, ions, and organic molecules in the clusters.** The point *r* = 0 is the center of mass of the cluster. Pure C_60_ in water (A), pure C_60_ in saline (B), C_60_ and acetophenone in water (C), C_60_ and benzaldehyde in water (D), C_60_ and benzyl alcohol in water (E), C_60_ and benzyl alcohol in saline (F), C_60_ and *m*-cresol in water (G), and C_60_ and toluene in saline (H). In graphs B, F and H, the scale of the left y-axis corresponds to C_60_ and organic molecules while the scale of the right y-axis corresponds to Na^+^ and Cl^−^. Plots from C to H highlight the fact that the outer region (shell) of the cluster is composed of organic molecules while the core is a mixture of C_60_ and organic molecules. For the spherically shaped clusters (C, D, E, H) a better delimitation of the core and shell regions is possible. The interface represents the contact region between shell and water and is defined by the point where the RDF of water (not shown) crosses the RDFs of organic molecules.
(TIF)

Table S1 **Description of the model systems simulated in this work.**
(DOCX)

Table S2 **Concentration of C_60_ in individual cell culture medium samples.** 2 mL of cell culture medium was extracted with 1 mL of toluene which was analyzed with LC-MS.
(DOCX)

Table S3 **Intensity average diameters of C_60_ aggregates in suspensions filtered through a 0.45 µm filter in individual samples.**
(DOCX)

Table S4 **ζ-potentials of C_60_ aggregates in suspensions filtered through a 0.45 µm filter in individual samples.**
(DOCX)

Table S5 **LDH release compared to positive control in individual unfiltered samples (%).**
(DOCX)

Table S6 **LDH release compared to positive control in individual filtered samples (%).**
(DOCX)

Table S7 **Concentration of IL-1β in individual unfiltered samples (pg mL^−1^).**
(DOCX)

Table S8 **Concentration of IL-1β in individual filtered samples (pg mL^−1^).**
(DOCX)

Table S9 **Concentration of TNF-α in individual unfiltered samples (pg mL^−1^).**
(DOCX)

Table S10 **Concentration of TNF-α in individual filtered samples (pg mL^−1^).**
(DOCX)
