# Supplementary figures and images for: Co-Exposure with Fullerene May Strengthen Health Effects of Organic Industrial Chemicals
Source: PLoS One. 2014 Dec 4;9(12):e114490. doi: 10.1371/journal.pone.0114490 (PMC4256445; doi:10.1371/journal.pone.0114490)

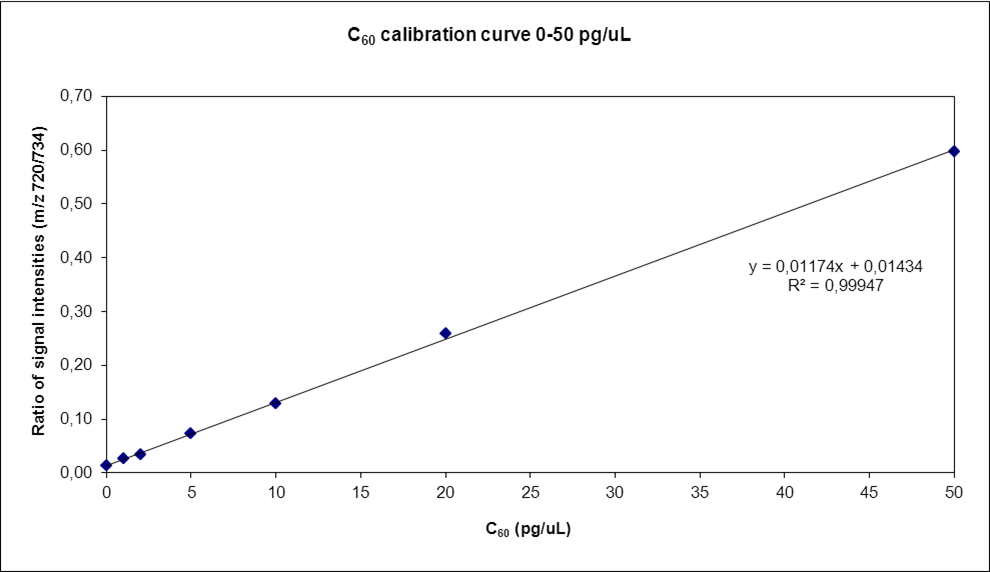

Supplement: Figure S1 — A calibration curve for the LC-MS analysis of the C60 concentration. (TIF) [file pone.0114490.s001.tif]

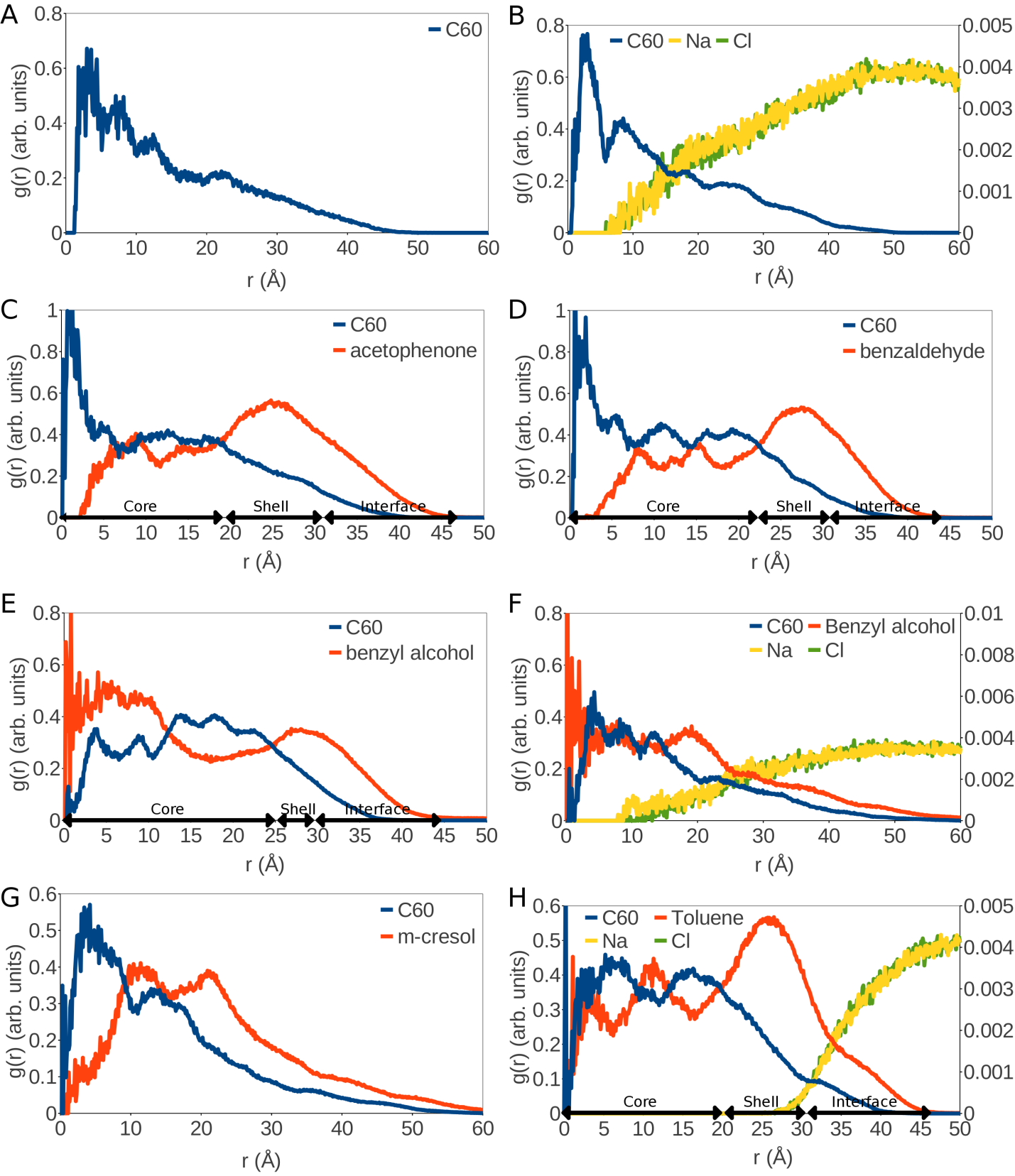

Supplement: Figure S3 — Radial distribution functions (RDFs) of C60, ions, and organic molecules in the clusters. The point r = 0 is the center of mass of the cluster. Pure C60 in water (A), pure C60 in saline (B), C60 and acetophenone in water (C), C60 and benzaldehyde in water (D), C60 and benzyl alcohol in water (E), C60 and benzyl alcohol in saline (F), C60 and m-cresol in water (G), and C60 and toluene in saline (H). In graphs B, F and H, the scale of the left y-axis corresponds to C60 and organic molecules while the scale of the right y-axis corresponds to Na+ and Cl−. Plots from C to H highlight the fact that the outer region (shell) of the cluster is composed of organic molecules while the core is a mixture of C60 and organic molecules. For the spherically shaped clusters (C, D, E, H) a better delimitation of the core and shell regions is possible. The interface represents the contact region between shell and water and is defined by the point where the RDF of water (not shown) crosses the RDFs of organic molecules. (TIF) [file pone.0114490.s003.tif]
