# Supplementary material for: Co-Exposure with Fullerene May Strengthen Health Effects of Organic Industrial Chemicals
Source: PLoS One. 2014 Dec 4;9(12):e114490. doi: 10.1371/journal.pone.0114490 (PMC4256445; doi:10.1371/journal.pone.0114490)
Supplement: Table S1 — Description of the model systems simulated in this work. (DOCX) [file pone.0114490.s004.docx]

**Table S1.** Description of the model systems simulated in this work.

| Simulated system | Solvent | Simulated time in each simulation | Number of independent simulations | Additional information |
| --- | --- | --- | --- | --- |
| 100 C_60_ | Water | 100 ns | 3 |  |
| 100 C_60_ | Saline | 100 ns | 1 |  |
| 100 C_60_ +  500 acetophenone | Water | 100 ns | 3 |  |
| 100 C_60_ +  500 benzaldehyde | Water | 100 ns | 3 |  |
| 100 C_60_ +  500 benzyl alcohol | Water | 100 ns  (or 200 ns, see right) | 3 | One simulation was continued up to 200 ns to observe cluster formation |
| 100 C_60_ +  500 benzyl alcohol | Saline | 100 ns | 1 |  |
| 100 C_60_ +  500 *m*-cresol | Water | 100 ns | 3 |  |
| 100 C_60_ +  500 toluene | Water | 100 ns | 3 |  |
| 100 C_60_ +  500 toluene | Saline | 100 ns | 1 |  |
| 100 C_60_ | Water |  |  | Used in free energy calculations |
| 100 C_60_ +  500 toluene | Water |  |  | Used in free energy calculations |
