# Supplementary material for: Co-Exposure with Fullerene May Strengthen Health Effects of Organic Industrial Chemicals
Source: PLoS One. 2014 Dec 4;9(12):e114490. doi: 10.1371/journal.pone.0114490 (PMC4256445; doi:10.1371/journal.pone.0114490)
Supplement: Table S2 — Concentration of C60 in individual cell culture medium samples. 2 mL of cell culture medium was extracted with 1 mL of toluene which was analyzed with LC-MS. (DOCX) [file pone.0114490.s005.docx]

**Table S2.** Concentration of C_60_ in individual cell culture medium samples. 2 mL of cell culture medium was extracted with 1 mL of toluene which was analyzed with LC-MS.

| Sample | Area (*m/z* 720) | Area (*m/z* 734) | Ratio of areas  (*m/z* 720/*m/z* 734) | C_60_ (*pg µL*^-1^) |
| --- | --- | --- | --- | --- |
| 1 | 41882 | 230386 | 0.182 | 14.262 |
| 2 | 29030 | 240204 | 0.121 | 9.072 |
| 3 | 40560 | 242278 | 0.167 | 13.037 |
| 4 | 28921 | 231875 | 0.125 | 9.402 |
| 5 | 34123 | 238589 | 0.143 | 10.960 |
