# Supplementary material for: Co-Exposure with Fullerene May Strengthen Health Effects of Organic Industrial Chemicals
Source: PLoS One. 2014 Dec 4;9(12):e114490. doi: 10.1371/journal.pone.0114490 (PMC4256445; doi:10.1371/journal.pone.0114490)
Supplement: Table S3 — Intensity average diameters of C60 aggregates in suspensions filtered through a 0.45 µm filter in individual samples. (DOCX) [file pone.0114490.s006.docx]

**Table S3.** Intensity average diameters of C_60_ aggregates in suspensions filtered through a 0.45 µm filter in individual samples.

| Suspension | Sample 1  Diameter (*nm*) | Sample 2  Diameter (*nm*) | Sample 3  Diameter (*nm*) |
| --- | --- | --- | --- |
| C_60_ | 202.1 | 188.4 | 214.0 |
| C_60_ + acetophenone | 213.1 | 225.5 | 243.1 |
| C_60_ + benzaldehyde | 210.4 | 214.4 | 213.2 |
| C_60_ + benzyl alcohol | 209.6 | 224.4 | 237.7 |
| C_60_ + *m*-cresol | 192.6 | 176.2 | 221.4 |
| C_60_ + toluene | 208.7 | 204.1 | 214.2 |
