# Supplementary material for: Co-Exposure with Fullerene May Strengthen Health Effects of Organic Industrial Chemicals
Source: PLoS One. 2014 Dec 4;9(12):e114490. doi: 10.1371/journal.pone.0114490 (PMC4256445; doi:10.1371/journal.pone.0114490)
Supplement: Table S4 — ζ-potentials of C60 aggregates in suspensions filtered through a 0.45 µm filter in individual samples. (DOCX) [file pone.0114490.s007.docx]

**Table S4.** ζ-potentials of C_60_ aggregates in suspensions filtered through a 0.45 µm filter in individual samples.

| Suspension | Sample 1  ζ-potential (*mV*) | Sample 2  ζ-potential (*mV*) | Sample 3  ζ-potential (*mV*) |
| --- | --- | --- | --- |
| C_60_ | -8.59 | -8.96 | -9.08 |
| C_60_ + acetophenone | -11.0 | -11.3 | -12.7 |
| C_60_ + benzaldehyde | -12.1 | -13.9 | -13.5 |
| C_60_ + benzyl alcohol | -11.4 | -12.5 | -14.2 |
| C_60_ + *m*-cresol | -11.0 | -12.9 | -12.3 |
| C_60_ + toluene | -11.2 | -13.0 | -13.5 |
