# Supplementary material for: Co-Exposure with Fullerene May Strengthen Health Effects of Organic Industrial Chemicals
Source: PLoS One. 2014 Dec 4;9(12):e114490. doi: 10.1371/journal.pone.0114490 (PMC4256445; doi:10.1371/journal.pone.0114490)
Supplement: Table S5 — LDH release compared to positive control in individual unfiltered samples (%). (DOCX) [file pone.0114490.s008.docx]

**Table S5.** LDH release compared to positive control in individual unfiltered samples (%).

| Exposure agent | Sample 1  LDH release (*%*) | Sample 2  LDH release (*%*) | Sample 3  LDH release (*%*) |
| --- | --- | --- | --- |
| C_60_ | 0.3 | 2.5 | 1.2 |
| Acetophenone | 16.8 | 49.2 | 29.6 |
| C_60_ + acetophenone | 28.9 | 59.3 | 34.3 |
| Benzaldehyde | 29.2 | 61.9 | 38.2 |
| C_60_ + benzaldehyde | 44.8 | 70.2 | 55.9 |
| Benzyl alcohol | 19.7 | 53.5 | 30.4 |
| C_60_+ benzyl alcohol | 25.6 | 55.6 | 28.5 |
| *m*-cresol | 9.4 | 73.4 | 17.0 |
| C_60_ + *m*-cresol | 22.5 | 76.7 | 19.1 |
| Toluene | 0 | 2.1 | 0 |
| C_60_ + toluene | 12.1 | 2.8 | 0 |
