# Supplementary material for: Co-Exposure with Fullerene May Strengthen Health Effects of Organic Industrial Chemicals
Source: PLoS One. 2014 Dec 4;9(12):e114490. doi: 10.1371/journal.pone.0114490 (PMC4256445; doi:10.1371/journal.pone.0114490)
Supplement: Table S6 — LDH release compared to positive control in individual filtered samples (%). (DOCX) [file pone.0114490.s009.docx]

**Table S6.** LDH release compared to positive control in individual filtered samples (%).

| Exposure agent | Sample 1  LDH release (*%*) | Sample 2  LDH release (*%*) |
| --- | --- | --- |
| C_60_ | 3.3 | 0 |
| Acetophenone | 44.1 | 23.6 |
| C_60_ + acetophenone | 45.4 | 13.8 |
| Benzaldehyde | 59.9 | 29.3 |
| C_60_ + benzaldehyde | 57.1 | 31.3 |
| Benzyl alcohol | 45.5 | 31.5 |
| C_60_+ benzyl alcohol | 53.3 | 28.0 |
| *m*-cresol | 71.1 | 18.0 |
| C_60_ + *m*-cresol | 66.0 | 12.5 |
| Toluene | 0 | 0 |
| C_60_ + toluene | 0 | 0 |
