# Supplementary material for: Co-Exposure with Fullerene May Strengthen Health Effects of Organic Industrial Chemicals
Source: PLoS One. 2014 Dec 4;9(12):e114490. doi: 10.1371/journal.pone.0114490 (PMC4256445; doi:10.1371/journal.pone.0114490)
Supplement: Table S7 — Concentration of IL-1β in individual unfiltered samples (pg mL−1). (DOCX) [file pone.0114490.s010.docx]

**Table S7.** Concentration of IL-1β in individual unfiltered samples (pg mL^-1^).

| Exposure agent | Sample 1  IL-1β (*pg mL*^-1^) | Sample 2  IL-1β (*pg mL*^-1^) |
| --- | --- | --- |
| None | 41.1 | 59.1 |
| C_60_ | 94.4 | 44.7 |
| Acetophenone | 51.0 | 104.1 |
| C_60_ + acetophenone | 67.8 | 95.1 |
| Benzaldehyde | 1651.1 | 900.6 |
| C_60_ + benzaldehyde | 1813.6 | 1240.4 |
| Benzyl alcohol | 66.0 | 38.8 |
| C_60_+ benzyl alcohol | 90.8 | 69.4 |
| *m*-cresol | 23.8 | 17.6 |
| C_60_ + *m*-cresol | 29.0 | 22.1 |
| Toluene | 58.4 | 32.4 |
| C_60_ + toluene | 64.4 | 50.1 |
