# Supplementary material for: Co-Exposure with Fullerene May Strengthen Health Effects of Organic Industrial Chemicals
Source: PLoS One. 2014 Dec 4;9(12):e114490. doi: 10.1371/journal.pone.0114490 (PMC4256445; doi:10.1371/journal.pone.0114490)
Supplement: Table S8 — Concentration of IL-1β in individual filtered samples (pg mL−1). (DOCX) [file pone.0114490.s011.docx]

**Table S8.** Concentration of IL-1β in individual filtered samples (pg mL^-1^).

| Exposure agent | Sample 1  IL-1β (*pg mL*^-1^) | Sample 2  IL-1β (*pg mL*^-1^) |
| --- | --- | --- |
| None | 28.2 | 58.3 |
| C_60_ | 40.6 | 44.0 |
| Acetophenone | 43.2 | 39.1 |
| C_60_ + acetophenone | 68.0 | 92.0 |
| Benzaldehyde | 924.6 | 737.6 |
| C_60_ + benzaldehyde | 1252.4 | 1126.2 |
| Benzyl alcohol | 47.4 | 31.7 |
| C_60_+ benzyl alcohol | 47.7 | 38.3 |
| *m*-cresol | 20.8 | 18.1 |
| C_60_ + *m*-cresol | 16.6 | 17.8 |
| Toluene | 34.6 | 18.5 |
| C_60_ + toluene | 32.7 | 34.6 |
