# Supplementary material for: Co-Exposure with Fullerene May Strengthen Health Effects of Organic Industrial Chemicals
Source: PLoS One. 2014 Dec 4;9(12):e114490. doi: 10.1371/journal.pone.0114490 (PMC4256445; doi:10.1371/journal.pone.0114490)
Supplement: Table S9 — Concentration of TNF-α in individual unfiltered samples (pg mL−1). (DOCX) [file pone.0114490.s012.docx]

**Table S9.** Concentration of TNF-α in individual unfiltered samples (pg mL^-1^).

| Exposure agent | Sample 1  TNF-α (*pg mL*^-1^) | Sample 2  TNF-α (*pg mL*^-1^) |
| --- | --- | --- |
| None | 4.2 | 15.9 |
| C_60_ | 7.8 | 9.5 |
| Acetophenone | 3.5 | 25.9 |
| C_60_ + acetophenone | 3.5 | 12.7 |
| Benzaldehyde | 103.4 | 309.7 |
| C_60_ + benzaldehyde | 124.6 | 191.1 |
| Benzyl alcohol | 11.3 | 2.8 |
| C_60_+ benzyl alcohol | 8.8 | 5.6 |
| *m*-cresol | 17.7 | 19.1 |
| C_60_ + *m*-cresol | 20.9 | 25.9 |
| Toluene | 11.7 | 4.6 |
| C_60_ + toluene | 8.5 | 7.0 |
