# Supplementary material for: Co-Exposure with Fullerene May Strengthen Health Effects of Organic Industrial Chemicals
Source: PLoS One. 2014 Dec 4;9(12):e114490. doi: 10.1371/journal.pone.0114490 (PMC4256445; doi:10.1371/journal.pone.0114490)
Supplement: Table S10 — Concentration of TNF-α in individual filtered samples (pg mL−1). (DOCX) [file pone.0114490.s013.docx]

**Table S10.** Concentration of TNF-α in individual filtered samples (pg mL^-1^).

| Exposure agent | Sample 1  TNF-α (*pg mL*^-1^) | Sample 2  TNF-α (*pg mL*^-1^) |
| --- | --- | --- |
| None | 4.2 | 24.8 |
| C_60_ | 3.9 | 23.1 |
| Acetophenone | 4.2 | 7.4 |
| C_60_ + acetophenone | 5.6 | 32.0 |
| Benzaldehyde | 125.6 | 369.8 |
| C_60_ + benzaldehyde | 140.3 | 597.5 |
| Benzyl alcohol | 7.0 | 3.9 |
| C_60_+ benzyl alcohol | 3.9 | 4.9 |
| *m*-cresol | 15.6 | 15.2 |
| C_60_ + *m*-cresol | 18.8 | 30.6 |
| Toluene | 4.2 | 6.3 |
| C_60_ + toluene | 4.9 | 7.8 |
